# Karyotyping Human Chromosomes by Optical and X-Ray Ptychography Methods

**DOI:** 10.1016/j.bpj.2014.11.3456

**Published:** 2015-02-03

**Authors:** Laura Shemilt, Ephanielle Verbanis, Joerg Schwenke, Ana K. Estandarte, Gang Xiong, Ross Harder, Neha Parmar, Mohammed Yusuf, Fucai Zhang, Ian K. Robinson

**Affiliations:** 1 London Centre for Nanotechnology, University College London, London, United Kingdom; 2 Research Complex at Harwell (RCaH), Rutherford Appleton Laboratory, Harwell Oxford, Didcot, United Kingdom; 3 Advanced Photon Source, Argonne National Laboratory, Argonne, Illinois

## Abstract

Sorting and identifying chromosomes, a process known as karyotyping, is widely used to detect changes in chromosome shapes and gene positions. In a karyotype the chromosomes are identified by their size and therefore this process can be performed by measuring macroscopic structural variables. Chromosomes contain a specific number of basepairs that linearly correlate with their size; therefore, it is possible to perform a karyotype on chromosomes using their mass as an identifying factor. Here, we obtain the first images, to our knowledge, of chromosomes using the novel imaging method of ptychography. We can use the images to measure the mass of chromosomes and perform a partial karyotype from the results. We also obtain high spatial resolution using this technique with synchrotron source x-rays.

## Introduction

The macroscopic structural property of mass can be used as a sensitive way to analyze differences between chromosomes and monitor changes that they may undergo during the cell cycle. The human genome is divided among 44+X+Y chromosomes, where each pair of chromosomes contains a different number of basepairs. The number of basepairs should not vary and therefore mass of the DNA contribution to the chromosomes can be well estimated. However, there are other structural components to the chromosomes such as the proteins (1), which may vary during the cell cycle as chromosomes replicate and change shape.

Early in their discovery it was found that chromosomes could be differentiated by size and, with the addition of stains, could be further identified by a unique banding pattern (2). This discovery led to the identification and sorting of chromosomes into a karyotype, which is still routinely used in the examination of chromosomes in clinical cytology laboratories. Human chromosomes are sorted by size and are given a number where 1 is the largest. The traditional karyotyping method uses chemical staining methods (such as G-banding) to highlight the position of the more condensed euchromatin and decondensed heterochromatin in the overall morphology of chromosomes. The stained chromosomes are measured with a visible light microscope; however, newer methods such as fluorescence microscopy are beginning to be used in hospitals to screen patients. These more advanced methods, e.g., m-FISH (multicolor fluorescence in situ hybridization), can be used to karyotype chromosomes in complex cases (3).

Nonimaging methods of karyotyping compare the number of basepairs in each chromosome and sort them accordingly. Chromosomes are an easily quantifiable object as each one contains a specific number of basepairs that is directly related to chromosome size therefore providing broad structural information that can be used for identification. The technique of Shotgun Sequencing is commonly used to analyze the genetic code and has successfully found the number of basepairs per chromosome with 90% accuracy (4). Shotgun sequencing is predominately used to look at euchromatin, as heterochromatin cannot be sequenced by widely used methods; however, it has been achieved in *Drosophila* (5). The number of basepairs, therefore, is calculated from predominately the euchromatin contribution in the chromosomes. Hence, a karyotype is performed using only partial information from the chromosomes.

A widely used way to sort chromosomes according to heterochromatin and euchromatin contribution is flow cytometry. This technique transports individual chromosomes stained with two dyes, one A-T specific, the other G-C specific, through a laser source, using a fluid. Both dyes are excited by the laser and the intensity of the emitted fluorescence is measured. The number of basepairs of G-C and A-T is assumed to be proportional to the emitted fluorescence. This karyotyping technique has been applied in evolutionary biology where similarities between chromosome structures of birds and reptiles can be easily seen in the similarities between their A-T and G-C content, which cannot be identified with other forms of chromosome analysis (6). This work shows the potential of information that can be obtained by performing karyotyping using information from the whole chromosome not just from the euchromatin contribution or placement of banding structures. Flow cytometry has been applied in karyotyping human chromosomes, however does not have sufficient resolution to distinguish chromosomes 9–12 in humans (7).

In this study, we apply ptychography (a phase retrieval imaging method) to the problem of karyotyping chromosomes. Phase retrieval imaging techniques are being developed to provide a method of imaging that avoids the use of lenses. These methods obtain images from diffraction measurements by recovering the nonmeasured phase using algorithms. The resulting complex image contains an accurate measure of the phase shift of the electromagnetic wave passing through the object at every point in the sample. The phase of the wave passing through the object is governed by the interaction between the electrons and the electromagnetic wave therefore the phase information is directly proportional to the electron density. Hence, this phase-contrast method produces an electron density map of the sample (8), from which the mass density can be directly calculated from a two-dimensional image. The mass information can then be used to sort the chromosomes by a quantifiable structural variable. We use the capability of doing mass measurements with ptychography to show a novel way, to our knowledge, of karyotyping chromosomes. The advantage of this method of karyotyping is that it takes into account the contribution from both the DNA and the proteins in the chromosomes and can be achieved without using invasive staining methods.

The imaging method of ptychography uses scanning coherent illumination (probe) and records diffraction patterns at overlapping positions (9). The high redundancy of the data allows the phase, lost during the measurement of the diffraction pattern, to be found by computerized algorithms. These algorithms iterate between real and reciprocal space applying constraints in both spaces until a solution is reached. A more detailed explanation of the ptychography method is given in the Theory and Implementation section.

In this study, we image a classical spread of chromosomes using ptychography with an optical laser source to provide a quantitative measure of mass density of isolated chromosomes. These measurements provide information on relative masses of chromosomes and therefore can be compared with other karyotyping techniques that use quantitative structural information to sort chromosomes such as volume measurements made with confocal microscopy and flow cytometry studies. By identifying the chromosomes measured with ptychography and confocal microscopy with m-FISH, we can compare our results to a flow-cytometry of human chromosomes performed in (10). We show that ptychography has sufficient resolution to karyotype chromosomes by their relative mass.

In the second part of this study, we image chromosomes by ptychography using a synchrotron radiation source of x-rays to try to increase the resolution of the imaging technique. High energy x-rays from synchrotron sources have a wavelength of 0.1–0.5 nm and therefore have the potential to provide a much higher resolution than laser-source ptychography (wavelength 400 nm). However, imaging with an x-ray source provides its own challenges such as the low image contrast due to the weak scattering of x-ray by proteins and reducing the radiation damage to the sample. Chromosomes have been successfully measured with coherent diffraction imaging, a closely related lensless imaging technique (11). Here, we show the first attempts, to our knowledge, of chromosomes measured with x-ray ptychography.

### Theory and Implementation

#### Ptychography algorithms

To retrieve the lost phase from the diffraction measurement the diffraction data must be correctly overdetermined following the criteria outlined in (12).

Ptychography is a lensless imaging technique that uses a scanning illumination function (probe) and exploits the overlap between adjacent positions to solve the following condition:

(1) $$\psi =P\left(\vec{r}\right)O\left(\vec{r}\right),$$

where ψ is the exit wave field from the sample, which is factorized into a contribution from the probe, $P\left(\vec{r}\right)$, and the object, $O\left(\vec{r}\right)$, where $\vec{r}$ is the position vector. The magnitude squared of the Fourier transform of this exit wave is captured on the detector, placed in the optical far field. The two functions *P* and *O* are uniquely defined by the condition that *P* is the same for all positions and *O* is the part that varies.

The following algorithms are used to retrieve the probe and object function from the measured intensity of the exit wave field. The extended ptychographic iterative engine (ePIE) (13) and the difference map algorithm (DM) (14) iterate between real and reciprocal space applying constraints in each. In reciprocal space the diffraction amplitude estimate is replaced by the measured diffraction amplitude, $\sqrt{I\left(\vec{q}\right)}$. This is usually referred to as the modulus constraint:

(2) $$\psi^{\prime }=\sqrt{I\left(\vec{q}\right)}\frac{ℑ\left\{\psi \right\}}{\left|ℑ\left\{\psi \right\}\right|},$$

where ℑ{ψ} is the Fourier transform of the exit wave field. In this constraint the estimated phase is kept but the estimated amplitude is replaced with the measured.

In real space the DM and ePIE algorithms update the probe and object function using different approaches, for more details see (13,14). The ePIE algorithm updates all the positions sequentially, whereas DM updates all positions in parallel.

The basic experimental setup for ptychography has a sample placed in line with a coherent source. A detector is placed in the far field to measure the intensity of the diffraction pattern from the sample. The sample is scanned through the beam using high precision translation stages and a diffraction pattern is taken at each overlapping position.

## Materials and Methods

### Sample preparation

#### Cell culture and chromosome isolation

Chromosomes were prepared from Yoruba lymphoblastoid (GM18507) cells that were at passage 4 following a protocol described in (15). Briefly, the cells were cultured in RPMI-1640 medium (Sigma Aldrich, UK) and were supplemented with 20% fetal bovine serum (Sigma Aldrich) and 1% l-glutamine, at a temperature 37°C in a 5% CO2 incubator. Once the cells were treated with Colcemid (Gibco BRL) at a final concentration of 0.2 *μ*gml−1, hypotonic treatment (0.075 MKCl) at 37°C for 5 min was given, followed by fixing the sample in three changes of 3:1, methanol/acetic acid.

#### For x-ray source ptychography

Chromosomes were prepared for x-ray imaging according to a previously published protocol for chromosomes by Nishino et al. 2009 (11).The chromosome sample was fixed in glutaraldehyde and placed onto a silicon nitride window containing 150 *μ*M of SYBR gold stain. The sample was washed in water to remove residues of dye and then left to air dry. Chromosome preparations were verified by imaging using a Zeiss AxioZ2 fluorescence microscope with ISIS software. Chromosomes on the same membrane were stained with Platinum blue, a dye synthesized in-house following the protocol in (16), in a concentration 5 mM for 30 min and washed for 5, 10, and 15 min in water.

#### For laser-source ptychography

20 *μ*l of suspension was dropped from a height onto a glass slide. The chromosomes were left to air dry. The chromosomes were stained with 0.1 *μ*g/l 4′,6-diamidino-2-phenylindole and the quality and density of the chromosome spreads was checked with a Zeiss Axio-Z2 fluorescence microscope. After the ptychography was completed the slide was then used to prepare chromosomes for m-FISH by the following method:

#### m-FISH

Karyotyping by m-FISH was performed as recommended by the 24XCyte m-FISH probe kit manufacturer (MetaSystems, Germany, http://www.metasystems-international.com) and according to a previously published protocol (17). The whole chromosome painting probes are directly labeled with the five different fluorophores in a combinatorial labeling format to provide 24 distinct colors. The hybridization of the probe with the cellular DNA site was visualized by means of fluorescence microscopy. The m-FISH images were analyzed using the MetaSystem’s ISIS m-FISH software.

#### Confocal microscopy

The volumes and areas of the chromosomes were measured with an Olympus LEXT-OLS4000. The volumes of the chromosomes were measured using the software package from the microscope to threshold an area from the background by height and then measure the volume in that region. Confocal microscopy is sensitive to the tilting of the sample substrate therefore the threshold is a baseline at the substrate level from which the heights of the sample are measured.

#### Laser-based ptychography

**Figure 1:**
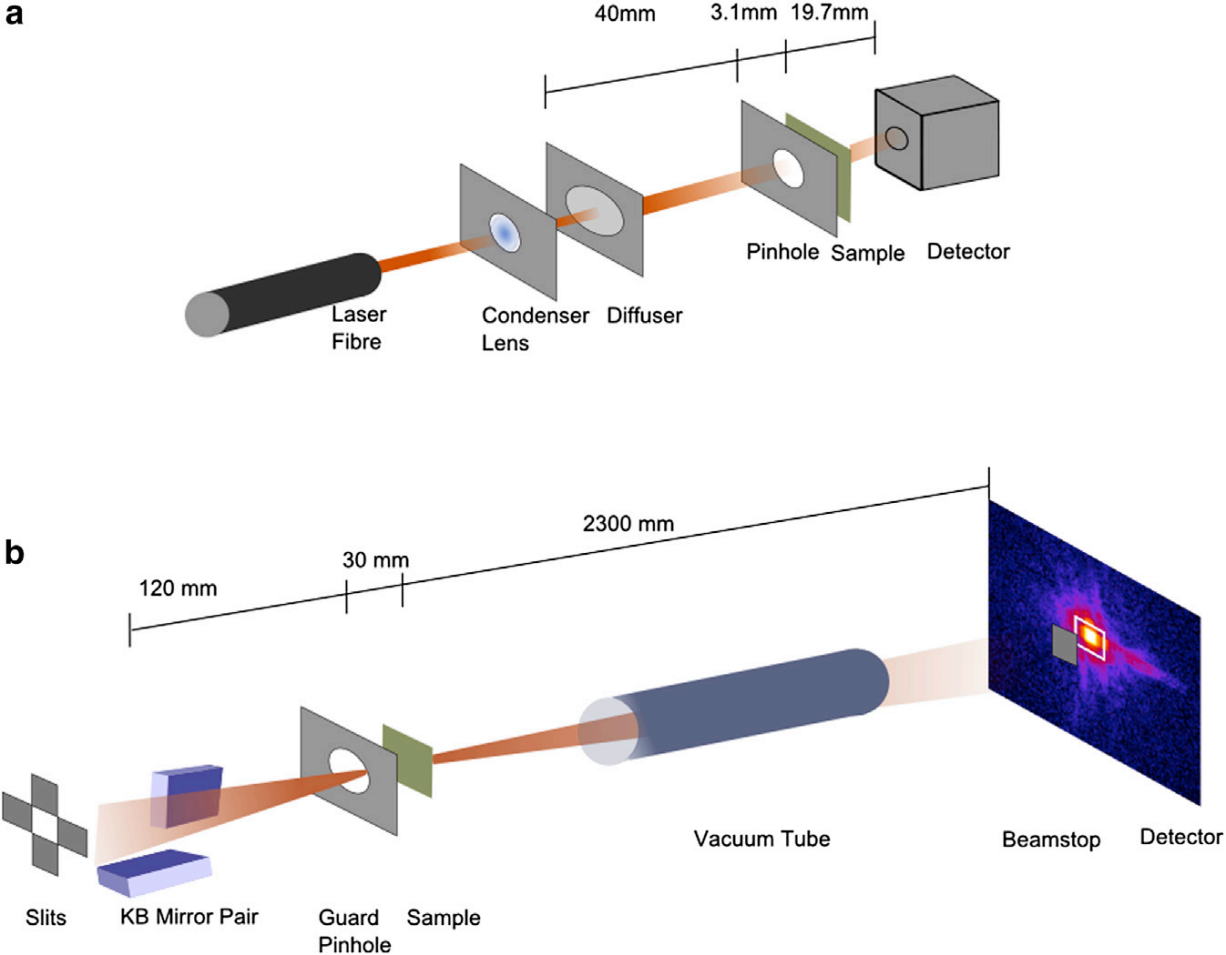
(*a*) Diagram of the laser ptychography setup. A diffuser is used to increase the angular divergence of the beam. The illumination is selected with a pinhole placed close to the sample. (*b*) Schematic of the x-ray ptychography setup. The illumination is focused on the sample with K-B mirrors and the signal hits the detector in the far field. A vacuum tube is used to reduce air scatter. To see this figure in color, go online.

For the chromosome karyotyping experiment, ptychography was performed with a laser source of wavelength λ = 406 nm (Fig. 1 ). A Thorlabs diffuser of 1° was placed 40 mm before a 0.5 mm pinhole to form the illumination probe. The use of a diffuser increases the resolution of the system by expanding the angular divergence of the probe and hence filling the detection numerical aperture. The sample was 3.1 mm behind the pinhole. An Andor sCMOS detector, with a 2048 × 2048 pixel array and pixel size 6.5 *μ*m^2^ was placed 19.7 mm behind the sample. The sample was scanned through the beam using a round region of interest scan pattern designed to remove the pathology of a typical raster scan (18). The step size was 0.1 mm and a 1 × 1 mm^2^ field of view of the sample was obtained. Images were reconstructed using 300 iterations of the ePIE algorithm from a 2048 × 2048 array.

#### Synchrotron-based ptychography

Experiments on chromosomes were carried out at the 34-ID-C beamline, Advanced Photon Source, Argonne National Lab, IL. Ptychography was performed using a photon energy of 5.5 keV, with a dwell time of 10 s per point. The scan time was ∼2 h per chromosome. The illumination was selected by an exit slit and focused onto the sample by a K-B mirror pair producing a beam of radius 300 nm at the focus. A single photon counting detector, model Timepix, was placed at 2.3 m from the sample. An attenuating beamstop of 200 *μ*m thick silicon was placed in front of the detector to avoid damage to the detector by the direct beam. As reported in (19), the central beam is only attenuated and therefore the signal measured under the beamstop can be reproduced with a multiplication of a calculated attenuation factor. Blocking the central part of the image would degrade the convergence of the algorithms (20). A round region of interest scan with a field of view of 4 *μ*m and step size of 0.25 *μ*m was used. Images were reconstructed using 10 iterations of ePIE then 480 iterations DM then 10 iterations ePIE, the probe function was updated after 10 iterations. It was found that this combination of ePIE and DM algorithms produced the best reconstruction. The DM algorithm is used to break the stagnation that sometimes occurs with ePIE by searching a wider solution space. The ePIE algorithm is used at the start and the end to speed up convergence to the solution minima. In this study each data set required a unique algorithm combination to obtain the highest resolution image.

#### Analyzing the phase

In this section, we are analyzing phase images, which are discretized into pixels, therefore we will adopt the following notation convention. The position vector can be expressed in a discrete way as the number of unit vectors, in this case pixel edges, $\vec{r}=\left(x,y\right)$, therefore we will express the functions dependent on $\vec{r}$ in terms of (x,y). The solution to the object function, *O* from Eq. 1 is a complex valued wave field that can be expressed as

(4) $$O=A\left(x,y\right)e^{-i\phi \left(x,y\right)},$$

where A(x,y) is the amplitude of the wave function, and the phase is given by ϕ(x,y).

The two-dimensional image is the projection of the three-dimensional density of the object for which the phase through each point ϕ(x,y) is related to the projected mass density dm/dxdy by the following:

(5) $$\phi \left(x,y\right)=\frac{2\pi \left(n-n_{0}\right)}{\lambda \rho }\frac{dm}{dxdy},$$

where *n* is the refractive index of the object, $n_{0}$ is the refractive index of the background, λ is the wavelength, and ρ is the density of the material. In the case of our experiment the background is air that has a refractive index $n_{0}=1$. In the case of visible light the excess density, i.e., the difference between the density of the material and the background, is proportional to $n-n_{0}$. For the case of an object in air the density of the material will be proportional to n−1 allowing this to be cancelled by the density in the denominator. The refractive index for x-ray wavelengths is expressed as n=1+δ−iβ, where the *δ* is related to the scattering and *β* relates to the absorption properties of the material. In the case of x-rays the phase is related to the *δ* part of the refractive index so Eq. 5 simplifies to

$$\phi \left(x,y\right)=\frac{2\pi \delta }{\lambda \rho }\frac{dm}{dxdy}.$$

The same analysis can be performed on images obtained with x-ray ptychography using this expression for the phase.

The mass of the chromosomes is found by integrating the phase across the chromosome area:

(6) $$m=\int dm=\frac{\lambda \rho }{2\pi \left(n-n_{0}\right)}\iint \phi \left(x,y\right)dxdy.$$

Because the image is in the form of discrete pixels the integral can be written as the following sum:

(7) $$m=\frac{\lambda \rho }{2\pi \left(n-n_{0}\right)}\sum_{n}\phi_{n}p,$$

where $\phi_{n}$ is the phase value of the image at pixel *n* and p is the pixel size.

To calculate the absolute mass for chromosomes an estimate of the density and refractive index is made. An estimate of the refractive index of chromosomes is taken from an experiment on DNA microfilms of 0.5–5 *μ*m thickness (21). Chromosomes are typically 1–2 *μ*m thick and we have approximated for now that they are entirely composed of DNA. From the study of DNA films it was found that the refractive index of a 1.5 *μ*m thick film was *n* = 1.54 at a wavelength of *λ* = 632.8 nm. This wavelength is different from the *λ* = 406 nm used in our experiment so the refractive index will be slightly different. The rough proportionality between density and refractive index make our result insensitive to the actual values of the quantities. However, the most important quantity for the karyotype is the relative masses of the chromosomes and not their absolute mass, therefore, the estimates of refractive index and density do not need to be considered because they are constant factors.

Due to the small area over which the mass calculation is performed the choice of threshold and background values are carefully made as the addition of a single pixel to the selected area can dramatically change the mass estimation. To take this into account the masses are calculated by taking the mean of the mass calculated for a 1% change in threshold value.

## Results

**Figure 2:**
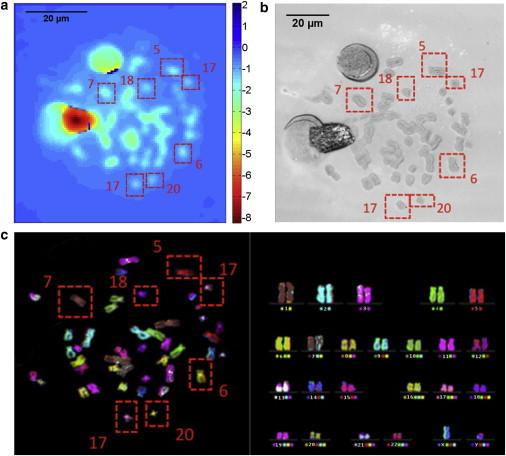
(*a*) Phase image of a chromosome spread with ptychography selected from a larger field of view. The small light green dots in the image are the chromosomes, the larger green and red circles are nucleus and debris (*b*) image of the same chromosome spread with confocal microscopy. Details such as chromatids and some of the smaller isolated chromosomes are seen. (*c*) The m-FISH karyotype of the same chromosome spread. The left image shows the chromosome spread with each chromosome showing its assigned color. The right image shows the sorting of the chromosomes by color into the karyotype. The chromosomes are identified by the number below each pair. The colored square on the right side of the number shows the combination of paints used to produce the colored circle on the left. The circle on the left shows the expected color of the chromosomes. To see this figure in color, go online.

First spreads of chromosomes were imaged with laser-source ptychography with sufficient resolution to identify isolated chromosomes. The image shows the phase of the chromosomes retrieved by ptychography (Fig. 2 *a*). The spatial resolution is estimated to be 1.8 *μ*m, which is sufficient to see larger individual chromosomes. In the center of the spread some chromosomes are overlapping or too close to be individually resolved or found through segmentation, therefore only isolated chromosomes were considered in the mass measurements. Two large circular objects are seen in the top left of the image, which are nuclei and debris. These were not analyzed as part of the chromosome study. The phase shift through the chromosomes represented in these images is significantly higher than the background allowing for the clear separation using the thresholding methods outlined in the Theory and Implementation section. The integrated phase was calculated for the seven isolated chromosomes, identified in the confocal and m-FISH images by red squares (Fig. 2, *b* and *c*). The mass was then calculated from the integrated phase (see section Theory and Implementation).

To verify the relative mass measurements calculated from the images produced by ptychography, the volumes of the chromosomes were measured with confocal microscopy. Volume should be linearly related to mass and therefore can be used to provide a direct comparison. The confocal microscope image (Fig. 2 *b*) has sufficient spatial resolution to show details of the chromatids and some of the smaller chromosomes that are not resolved with ptychography (Fig. 2 *a*).

To identify the chromosomes that were imaged with ptychography and confocal microscopy a standard karyotype was performed on the same spread with m-FISH. This fluorescence technique uses computer-generated colors from a coding scheme, which analyzes the emitted fluorescence from various pairs of five paints (see section Materials and Methods). The full karyotype (Fig. 2 *c*) shows the chromosomes ordered with their identifying number underneath. Left of this number a colored spot indicates the expected color from the technique and on the right the squares shows the number of fluorescent colors that were used to make the represented color. The m-FISH results show the expected color for the majority of the chromosomes, with the exception of 1 and 12. However, the observed centromere positions and chromosome size, indicate with confidence, that these chromosomes are 1 and 12. Appearance of the wrong color can be caused by the bleeding of the different dyes used in this technique. The chromosomes that were measured with ptychography, as shown in the red boxes, are all correctly identified by m-FISH, hence the chromosomes measured with confocal microscopy and ptychography can be identified by chromosome number.

**Figure 3:**
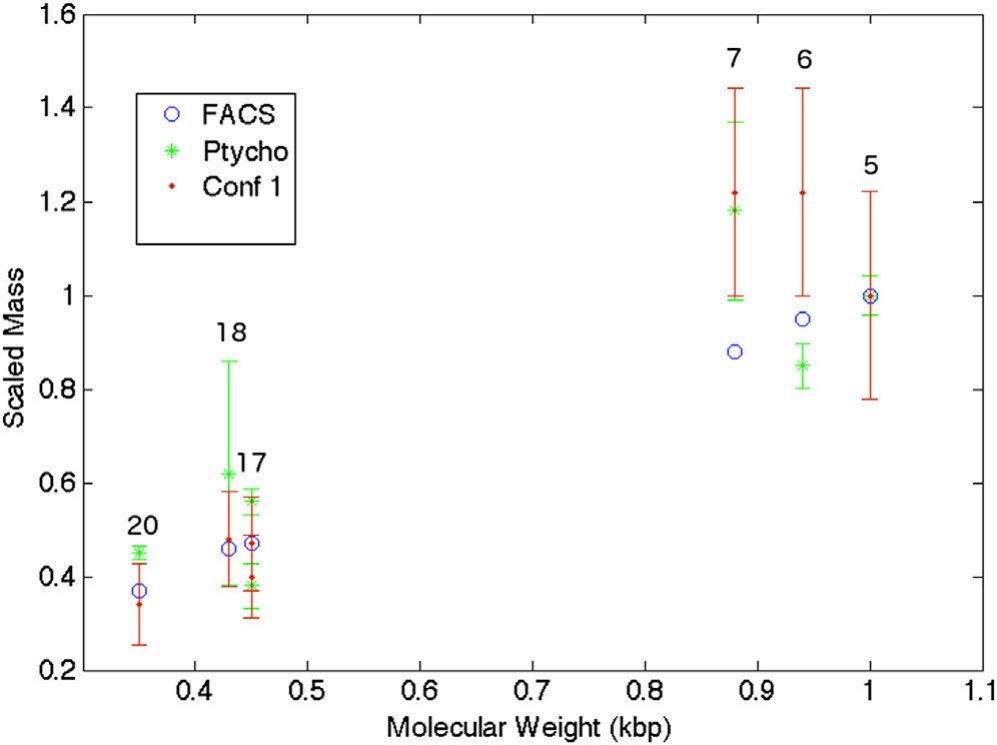
The mass-related values found by the various karyotyping methods are shown against molecular weight as found from sequencing (22). The numbers above the points are the chromosome identifying numbers. The masses are scaled relative to the mass of chromosome 5, the largest chromosome measured. Two chromosome 17s were measured by ptychography and are represented twice in the plot. The blue circles show the scaled fluorescence of FACS from (10). Scaled mass results from ptychography are shown by green stars. The scaled volumes from the confocal measurements are shown by the solid red dots. To see this figure in color, go online.

The relative mass and volume results can then be compared to the Molecular Weight as found by sequencing from (22). A further comparison can be made to the flow cytometry study of human chromosomes from (10). Masses calculated from each technique are scaled against the mass of chromosome 5, the largest chromosome measured by all three techniques. The relative mass of chromosomes measured by the methods of ptychography, confocal microscopy, and flow cytometry are plotted against Molecular Weight (Fig. 3). The error bars on the measurements are determined by the change in mass from a 1% change in threshold value.

The relative mass of the chromosomes shows a decrease with molecular weight, however, without any data between chromosome 7 and 17 the trend cannot be assumed to be linear. The smaller chromosomes (17–19) measured in our study show better agreement with the result from flow cytometry, whereas the larger chromosomes (6,7) deviate from the flow cytometry results. The range of relative masses is 0.08 among chromosomes 17–20 but is 0.29 between chromosomes (6,7).

There is also a difference between the sizes of the chromosomes measured with confocal microscopy and with ptychography. Chromosome 6 is measured to be larger than chromosome 5 with confocal microscopy but ptychography makes the mass of chromosome 6 smaller than that of chromosome 5. There is a similar disagreement between the sizes measured by the two techniques in chromosome 18. Both the confocal microscopy measurement and the ptychography measurement show chromosome 7 to be larger and heavier, respectively, than chromosome 5.

**Figure 4:**
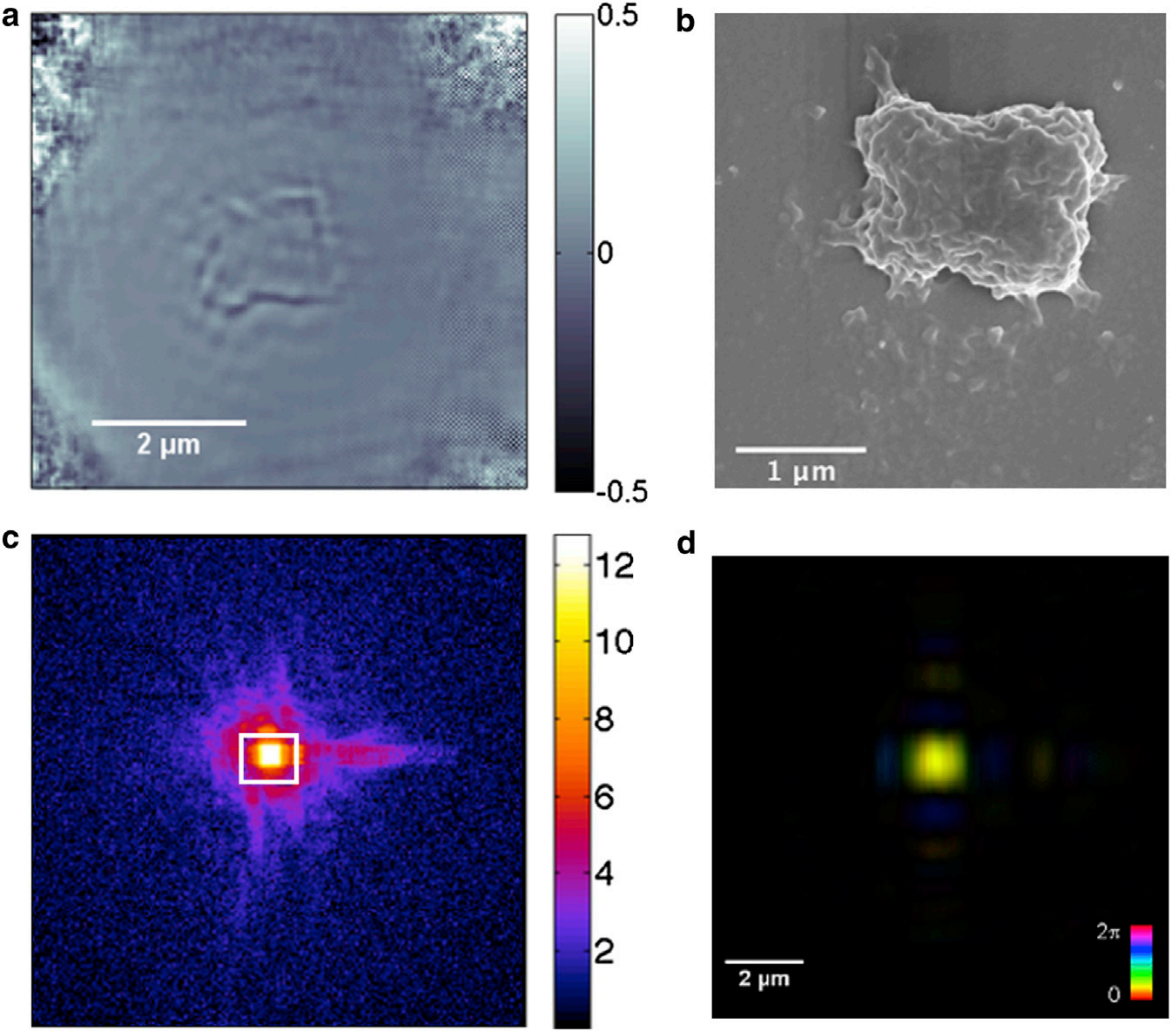
(*a*) Phase image of a chromosome from x-ray ptychography performed at 34-ID-C, APS. A ringing effect can be seen at the borders of the chromosome. (*b*) Scanning electron microscopy image of the chromosome image as ptychography, performed at UCL. (*c*) Diffraction pattern from the chromosomes, the white square shows where the beamstop is covering the central data. (*d*) Illumination retrieved from ptychography, where the phase information is given by the color and the amplitude information by the intensity. To see this figure in color, go online.

Although the laser ptychography is sufficient to calculate mass, an increase in resolution would certainly benefit the application, therefore x-ray-source ptychography was used to image human chromosomes. The recovered phase from the chromosome by ptychography is shown in Fig. 4 *a*. The outline of the chromosomes can be clearly identified in this image, however there is a strong ringing effect at the boundary of the object. These images are compared with scanning electron microscopy images of the same chromosomes obtained after imaging with ptychography (Fig. 4 *b*). It can be seen that retrieved images from ptychography represent well the gross morphology of the chromosomes; however, there is still details missing such as the individual chromatids that are probably caused by the ringing effects at the edges of the images.

These images are measured to a spatial resolution of 370 nm, which is much higher than the 1.8 *μ*m resolution of the laser ptychography setup. The resolution is calculated from the maximum scattering angle measured from the diffraction pattern (Fig. 4 *c*). Despite imaging with sufficient resolution to see individual chromosomes, the phase information cannot be easily separated from the background. This is caused by a rippling artifact around the boundary of the chromosome, which makes it difficult to establish a border around the chromosome object. It can also be seen that the majority of the phase shift occurs at the edges of the chromosome and the phase inside the main body of the chromosome is not much above the level of the background.

Ptychography also retrieves the illumination function (Fig. 4 *d*). The illumination shows the expected form from a K-B mirror pair used to define the probe in this experiment further validating the accuracy of the reconstruction. The illumination is displayed such that color represents phase and intensity represents amplitude.

## Discussion

In comparing the relative sizes of the chromosomes a good agreement is found between the methods. From the flow cytometry experiment in (10), (*partially shown* in Fig. 3), there is a strong linear trend between molecular weight and number of basepairs. The masses measured in this study by confocal microscopy and ptychography show a decrease in mass with molecular weight but there is not enough data to fully establish a linear trend. The confocal microscopy has a higher resolution and therefore could be used to measure the volume of chromosomes between 7 and 17 to verify linearity. Where there is considerable overlap, it is difficult to separate the volumes of the two chromosomes with this technique. Further investigation could use confocal microscopy to measure chromosomes and establish linearity.

In the spread, a pair of chromosomes 17s was successfully imaged with a degree of accuracy to calculate their mass, however the calculated masses do not show a good agreement. The cause of this discrepancy could be due to the spatial resolution of the confocal and ptychography measurement techniques. In ptychography the spatial resolution is such that each chromosome is formed of 4 or 5 pixels of phase, therefore detection of phase through a single pixel can change the mass greatly, giving very high error margins to the technique. Similar to confocal microscopy the resolution of an optical slice is 60 nm, about half the approximate thickness of an air-dried chromosome, prepared with these methods.

It can be seen from these results that the limitation in applying ptychography to karyotyping is the resolution that can be achieved with a laser. The spatial resolution is only sufficient to see large isolated chromosomes, the smaller chromosomes are represented in the image over 2–3 pixels. This produces large errors on the mass measurements as the addition of 1 pixel into the calculation can cause large changes in the mass. However, it has been shown that even with limited resolution it is possible to start sorting the chromosomes by mass. Greater spatial resolution would greatly improve the accuracy of the mass calculation. To achieve this, a high energy x-ray source, which has a far smaller wavelength than visible light, has been used.

The images of the chromosomes measured by x-ray ptychography shown here are the first attempts, to our knowledge, with this technique and show a promising start that can be improved with better experimental methods. In our experiments, it was found to be difficult to measure the integrated phase in the resulting images due to rippling artifact at the edges of the chromosome image. The rippling effects can often be reduced by propagating the entire wave field as retrieved by ptychography. This was attempted without improvement to the image.

There are two possible causes of ringing artifact: the first is the beamstop, (shown as the *square area in the center* of Fig. 4 *c*), suppressing the low frequency information and therefore acting like a high-pass filter. This filtering causes sharp contrast at the edges of the chromosome where the high frequency information is represented. The second could be caused by the presence of higher wavelengths in the illumination source. Ptychography is performed with monochromatic sources, which in a synchrotron is produced by a monochromator and mirror. If these are not correctly aligned or used at the end of their energy range, higher wavelengths can pass through the monochromator. The 34-ID-C beamline operates in the energy range of 5–11 keV, and measuring at 5.5 keV is nearing the edge of this limit. Recent developments in ptychography show that it is possible to extract the two modes produced by the harmonics (23). This type of reconstruction was attempted on the data but without improvement to the overall image. The weak scattering nature of the sample may reduce the effectiveness of this technique.

When measuring biological samples with x-rays, radiation damage is an important consideration. In this study samples were dehydrated and treated with glutaraldehyde to reduce the effects of radiation damage on the sample. Damage to the sample affects the high resolution information first and can be seen by the fading of the high order diffraction over time (24). The diffraction data taken in this study showed no such fading, however, the sample will have undergone some damage after prolonged x-ray exposure.

## Conclusions

This study shows first attempts, to our knowledge, of imaging human metaphase chromosomes with ptychography. The chromosomes were imaged with sufficient quality by laser-source ptychography that a partial karyotype can be performed using the mass measured with this technique. The spatial resolution of the laser-source ptychography is limiting the measurements of the smaller chromosomes, therefore extending the resolution of this technique would be beneficial for better mass measurements.

We have shown that synchrotron source ptychography can produce higher spatial resolution images of chromosomes. The artifacts in the images are too great for the mass to be accurately calculated for these images, however with improvements to the experiments these can be reduced. To improve the images the weak scattering power of the sample must be addressed. This can be achieved in an experimental way by modifying the setup to reduce air scatter and radiation damage by moving toward a cryo-vacuum sample chamber. Efforts on the sample preparation including staining with a contrast enhancing heavy metal dye could increase the scattering from the chromosomes and hence improve the resolution and quality of the retrieved images. For biological samples it would be beneficial to measure at lower energies where the scattering of the light elements is greater. Considering recent efforts in soft x-ray ptychography (25,26), measuring the chromosomes at a low energy could provide high-resolution images with good phase contrast to be used for karyotyping.
